# The role of body composition and appetite-regulating hormones in idiopathic central precocious puberty and their changes during GnRH analog therapy

**DOI:** 10.1007/s40618-024-02413-3

**Published:** 2024-06-19

**Authors:** G. Tarçin, E. Bayramoğlu, D. Güneş Kaya, H. Karakaş, K. C. Demirbaş, H. Turan, O. Evliyaoğlu

**Affiliations:** 1 Cerrahpaşa Faculty of Medicine, Department of Pediatric Endocrinology, Istanbul University-Cerrahpaşa, Istanbul, Turkey; 2 Cerrahpaşa Faculty of Medicine, Department of Pediatrics, Istanbul University-Cerrahpaşa, Istanbul, Turkey

**Keywords:** Body composition, Ghrelin, Leptin, Neuropeptide Y, Peptide YY, Precocious puberty

## Abstract

**Purpose:**

It was aimed to compare circulating levels of ghrelin, leptin, peptide YY (PYY), and neuropeptide (NPY) between girls with idiopathic central precocious puberty (ICPP) and prepubertal girls, as well as to evaluate alterations in these hormone levels and body composition during leuprolide acetate treatment in girls with ICPP.

**Methods:**

This prospective study was conducted on girls with isolated premature thelarche (IPT), girls with ICPP, and age-matched prepubertal controls. Anthropometric measurements, body composition analysis and appetite-regulating hormone level measurements were performed in each group and also at the 6th and 12th months of the leuprolide acetate treatment for the girls with ICPP.

**Results:**

Seventy-three girls participated in the study (24 girls with ICPP, 28 with IPT, and 21 prepubertal controls). No significant differences were observed in ghrelin, leptin, PYY, and NPY levels among the three groups. Leuprolide acetate treatment resulted in increased leptin, decreased PYY and NPY levels, and no significant changes in ghrelin. Despite no significant change in body mass index standard deviation score (BMI SDS), body fat percentage increased during treatment.

**Conclusion:**

While appetite-regulating hormones do not seem to directly contribute to precocious puberty pathogenesis, puberty blockade was shown to lead to altered levels of these hormones along with changes in body composition.

**Supplementary Information:**

The online version contains supplementary material available at 10.1007/s40618-024-02413-3.

## Introduction

The timing of pubertal onset is known to be intricately influenced by genetic, nutritional, environmental, and socioeconomic factors. However, despite decades of investigation, the mechanisms underlying the early activation of the Gonadotropin-releasing hormone (GnRH) axis, i.e., precocious puberty, have not yet been fully understood. Indeed, not just one, but several interacting mechanisms have been recognized to play a role [1, 2]. Among these mechanisms, nutrient and energy sensing systems are of particular interest.

As early as the 1970s, Frisch proposed the “critical weight hypothesis”, which suggested that a certain body fat depot was required to initiate normal reproductive function [3]. This hypothesis was confirmed by several studies showing a relationship between higher prepubertal body mass index (BMI) and earlier puberty onset and menarche [4–7]. Additionally, one factor considered as a cause of the secular trend toward earlier puberty is the ongoing global obesity epidemic [8]. In addition to obesity, body fat distribution has recently been associated with sexual development [9]. As it is evident that body composition and energy balance mechanisms are critical for the onset and development of puberty, the role of appetite-regulating hormones in this process has also been investigated recently.

Ghrelin and leptin function as counterbalancing regulators of energy balance by producing opposing effects. Ghrelin mainly serves as a signal of energy deficiency, with its concentrations increasing during fasting. Having receptors in the hypothalamic-pituitary–gonadal axis, it exerts a negative effect on luteinizing hormone (LH) pulsatility and sex steroid production [10]. Conversely, leptin is a satiety hormone secreted by adipocytes, and its circulating levels correlate with fat mass. Before puberty, leptin concentrations gradually increase with age, suggesting the presence of a leptin concentration threshold that triggers the onset of puberty. Administering this hormone to individuals with leptin deficiency or hypogonadotropic hypogonadism can promote pubertal development, and parallelly, when given to juvenile mice, it has been reported to induce precocious puberty. Although the mechanism of its action is not fully understood, it is believed to involve a direct impact of leptin on neuronal nitric oxide synthase neurons in the hypothalamic preoptic region, resulting in increased LH secretion [1, 10, 11].

Peptide YY (PYY), secreted by the intestines in response to food intake can cross the blood–brain barrier and inhibit hypothalamic neuropeptide Y (NPY) neurons through Y2 receptors, leading to reduced food intake in rodents. Additionally, PYY administration has been reported to inhibit GnRH secretion in male rats. However, data on the impact of PYY on the reproductive axis in humans are limited. Girls with anorexia nervosa, which is often associated with a hypothalamic hypogonadal state, have higher PYY levels than healthy peers. This suggests that elevated PYY may contribute to reduced food intake and potentially influence the reproductive axis [10, 12]. While it appears that GnRH neurons can directly sense changes in nutrient availability, indirect pathways may play a more significant role than direct GnRH sensing in the metabolic control of the HPG axis. In this context, the arcuate nucleus houses key components, such as proopiomelanocortin (POMC) and NPY/Agouti-related protein (AgRP) neurons, which are master regulators of energy balance. These neurons have been shown to directly affect GnRH excitability. Specifically, NPY/AgRP neurons influence food intake and metabolism with orexigenic actions, and their activation has been observed to reduce LH pulse frequency in gonadectomized mice [13].

In short, the relationship of both body composition and appetite-regulating hormones with puberty is evident, but studies regarding their association with precocious puberty have only recently been increasing, and there is very limited research on their changes during puberty blockade [14, 15]. In this study, it was aimed to compare the circulating levels of ghrelin, leptin, PYY and NPY between the girls with idiopathic central precocious puberty (ICPP) and prepubertal girls, and also to evaluate the alterations in the levels of these hormones during leuprolide acetate treatment.

## Material and methods

### Study design and creating study groups

This is a prospective study conducted on girls with premature thelarche and age-matched healthy prepubertal controls. The study included all girls referred to the pediatric endocrinology outpatient clinic between June 2021 and February 2022 due to breast development before the age of 8 years.

Once premature thelarche was confirmed by a pediatric endocrinologist, these girls underwent a series of investigations, including physical examination, biochemical tests, hand and wrist X-ray, pelvic ultrasound and luteinizing hormone-releasing hormone (LHRH) stimulation test to distinguish between ICPP and isolated premature thelarche (IPT). Girls diagnosed with precocious puberty underwent contrast-enhanced brain and pituitary MRI. Those found to have an organic pathology were excluded from the study, leaving only idiopathic cases.

After forming the patient groups, prepubertal healthy controls of similar ages to the patients were recruited from the general pediatric outpatient clinic. These controls included children who presented for a routine check-up or with concerns about height, weight, or pubertal development and were ultimately found to be free of any endocrinological disorders. Inclusion criteria for the controls included not having a chronic disease, not taking medication, and being at Tanner stage 1 based on the examination by a pediatric endocrinologist. Finally, three groups were formed: the girls with ICPP, the girls with IPT and prepubertal controls.

### Evaluation of the subjects

All girls underwent anthropometric measurements, including height and weight, using a stadiometer (Holtain Limited, Crymych, Wales) and an electronic scale (Tanita MC-780 MA, Tanita Corporation, Tokyo, Japan). Standard deviation scores (SDSs) for height, weight, and BMI were calculated according to the standards for Turkish children [16]. Pubertal stage was assessed based on the Tanner scale [17].

Girls with premature thelarche underwent initial biochemical tests, including serum follicle stimulating hormone (FSH), LH, estradiol, thyroid-stimulating hormone (TSH), and free thyroxine (fT4). Bone age was determined using the Greulich and Pyle method. A GnRH stimulation test was performed with a standard dose of 0.1 µg GnRH administered as an intravenous bolus. Blood samples for FSH and LH were collected just before the injection and at 30 min intervals until the 120th minute after the injection. A peak LH value of more than 5 IU/L was considered suggestive of puberty. In pelvic ultrasound, a uterine length of more than 35 mm and ovarian volumes above 2 cc were considered consistent with puberty [18].

Body composition analysis was conducted using the bioelectrical impedance method (Tanita MC-780 MA, Tanita Corporation, Tokyo, Japan). A dietitian obtained a 24 h dietary recall, and total caloric intake, carbohydrate, fat, and protein intake, as well as macronutrient ratios, were calculated using Ebispro for Windows (BeBiS v.9) (Stuttgart, Germany).

### Biochemical analysis

Blood samples were collected after an overnight fasting state, centrifuged, and stored at -80 °C until analysis for measurements of ghrelin, leptin, PYY, and NPY levels. The measurements were carried out using enzyme-linked immunoassay (ELISA) kits (Elabscience Biotechnology Co., Ltd, China for ghrelin, PYY, and NPY; DRG International, Inc., USA for leptin) with an absorbance microplate reader (BioTek ELx800, USA) following the manufacturer’s instructions. FSH, LH and estradiol levels were measured by electrochemiluminescence immunoassay (ECLIA).

### Treatment protocol and monitoring of the patients with precocious puberty

Girls diagnosed with ICPP were initiated on leuprolide acetate treatment. The patients were prescribed either 3.75 mg of leuprolide acetate every 28 days (Lucrin Depot® 3.75 mg, AbbVie, Tokyo, Japan) or 11.25 mg every 3 months (Lucrin Depot® 11.25 mg, AbbVie, Tokyo, Japan). The treatment approach followed a predetermined sequential method, with patients alternating between the two dosage options. After starting treatment, clinical evaluation, body composition analysis, blood sample collection for measuring appetite-regulating hormones, and a 24 h food intake record were repeated at the 6th and 12th months. Additionally, serum LH levels were measured 30 and 60 min after leuprolide acetate injection at these milestones. Pubertal suppression was defined as a stimulated LH level less than 4 IU/L [19].

### Statistical analysis

The statistical analyses were performed using the Statistical Package for Social Sciences (SPSS), version 27 (Chicago, Illinois). Data distribution was evaluated through histograms and the Shapiro–Wilk test. Data with a parametric distribution were presented as mean ± SDS, while data with a nonparametric distribution were presented as median (minimum–maximum). Comparisons among the three groups were conducted using either the Kruskal–Wallis test or one-way analysis of variance (ANOVA), depending on the data distribution. Parameters within the ICPP group at 0, 6, and 12 months of treatment were compared using either repeated measures ANOVA or the Friedman test, depending on the data distribution. In cases where the Friedman test yielded statistical significance, post-hoc tests were conducted using the Wilcoxon signed-rank test with Bonferroni correction for pairwise comparisons. A Spearman correlation analysis was employed to assess the relationships between non-normally distributed variables. A p-value less than 0.05 was considered statistically significant.

### Ethical considerations

This study was conducted in accordance with the principles of the Declaration of Helsinki. Approval was granted by the Ethics Committee of Istanbul University-Cerrahpaşa. Informed written consent was obtained from the parents of all subjects to participate in the study.

## Results

### Characteristics of the patient groups

A total of 73 girls were included in the study (24 girls with ICPP, 28 girls with IPT and 21 prepubertal controls). There were no statistically significant differences in terms of age, BMI SDS, among the three groups (Table 1).

**Table 1:** Comparisons of appetite-regulating hormone levels, the parameters of body composition analysis and 24 h food intake record among the three groups

	ICPP (n = 24)	IPT (n = 28)	Controls (n = 21)	*p*
Age (years)	7.6 ± 0.9	7.4 ± 0.9	7.9 ± 1.0	0.183
Appetite-regulating hormones				
Ghrelin (ng/mL)	7.96 (4.12–13.97)	8.66 (3.54–14.37)	7.91 (2.01–13.42)	0.461
Leptin (ng/mL)	2.88 (0.40–13.40)	2.95 (0.45–24.07)	3.85 (0.16–14.67)	0.980
PYY (pg/mL)	46.23 (9.74–161.27)	56.07 (17.60–136.83)	55.11 (27.36–124.63)	0.402
NPY (pg/mL)	2775.58 (692.18–14,349.27)	3065.63 (484.56–17,803.11)	2621.91 (252.89–11,339.69)	0.441
Body composition analysis				
BMI SDS	1.04 ± 0.78	1.05 ± 1.12	0.79 ± 1.43	0.681
Body fat mass (kg)	7.8 (4.4–22.7)	6.9 (2.7–26.5)	6.9 (3.6–15)	0.525
Body fat percentage (%)	24.5 (17.5–41.1)	25.0 (16.9–44.1)	27.0 (12.8–37.4)	0.982
Fat-free mas (kg)	23.5 (14.1–35.9)	21.4 (12.8–33.5)	21.3 (16.3–31.6)	0.139
Muscle mass (kg)	22.3 (13.3–34.1)	20.3 (12.1–31.8)	20.2 (15.4–30.0)	0.136
Truncal fat mass (kg)	3.4 (1.6–10.1)	3.2 (1.1–11.1)	3.1 (0.9–6.8)	0.659
Truncal fat percentage (%)	19.3 (10.6–35.6)	19.6 (10.3–37.1)	20.6 (6.0–32.1)	0.971
24 h food intake record				
Total caloric intake (kcal)	1012 (818–1348)	1168 (715–2320)	1139 (934–1827)	0.174
Carbohydrate intake (gr)	100.9 (83.9–170.5)	101.7 (61.2–341.8)	125.5 (66.4–219.3)	0.115
Fat intake (gr)	38.5 (32.4–83.6)	47.1 (21.8–84.6)	55.5 (25.7–79.1)	0.236
Protein intake (gr)	34 (27.2–59.1)	42.2 (16.1–93.2)	36.9 (19.9–54.1)	0.092
Macronutrient ratios				
Carbohydrate (%)	47 (27–63)	42 (29–61)	48 (26–68)	0.175
Fat (%)	37 (26–55)	47 (16–55)	40 (19–63)	0.125
Protein (%)	14 (11–18)	13 (9–30)	13 (8–18)	0.714

*BMI* *SDS* body mass index standard deviation score, *ICPP* idiopathic central precocious puberty, *IPT* isolated premature thelarche, *NPY* neuropeptide Y, *PYY* peptide YY

Out of the initial 24 patients diagnosed with ICPP, half of the patients were started on the monthly 3.75 mg form of leuprolide acetate, while the other half were initiated on the 3 month 11.25 mg form. However, due to a loss to follow-up by 4 patients, the study continued with a cohort of 20 ICPP patients, with 11 patients remaining on the 3.75 mg dosage and 9 patients on the 11.25 mg dosage.

### Comparisons of the study parameters among the groups

As shown in Table 1, there were no significant differences in ghrelin, leptin, PYY, and NPY levels among the three groups. Additionally, BMI SDS and all the parameters for body composition analysis and food intake records were similar among the three groups.

### Changes in the study parameters under leuprolide acetate treatment in the ICPP group

All 20 patients who continued the treatment until the endpoint of the study exhibited pubertal regression in their clinical examination and also according to the stimulated LH levels at both the 6th and 12th months, regardless of the form of leuprolide acetate used.

**Fig. 1:**
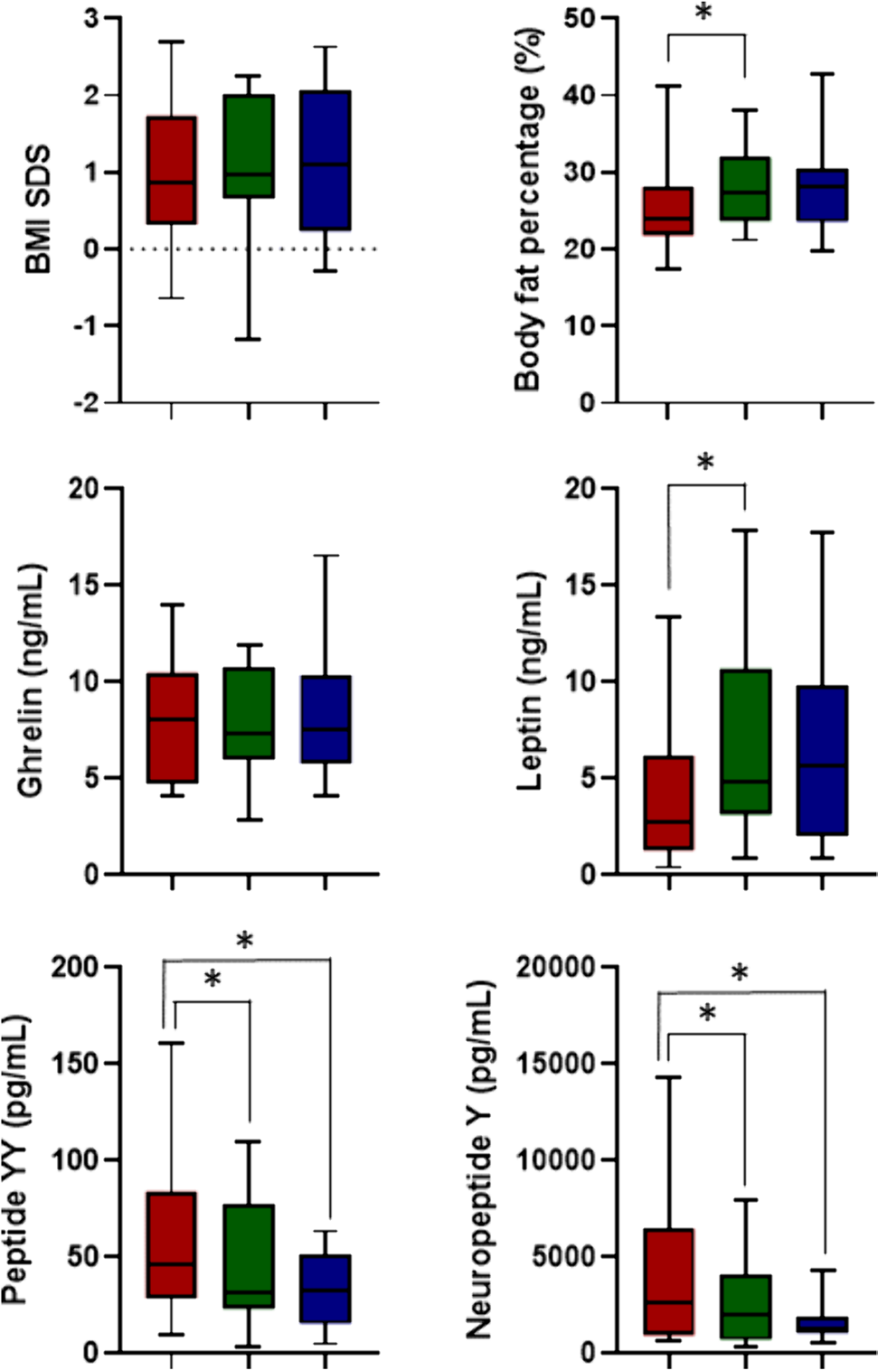
Box plots illustrating changes in appetite-regulating hormones and body composition at baseline (red), 6th (green), and 12th (blue) months of leuprolide acetate treatment in the ICPP group. *Statistically significant at a significance level of p < 0.017 after post-hoc pairwise comparison with Bonferroni correction

As shown in Table 2 and Fig. 1, during leuprolide acetate treatment, ghrelin levels did not show any significant changes. However, a significant increase in leptin, and a significant decrease in both NPY and PYY levels were found. Although BMI SDS did not alter significantly, there was a significant increase in body and truncal fat percentage. Finally, caloric intake, carbohydrate, fat and protein intake, as well as macronutrient ratios did not change significantly.

**Table 2:** Comparison of appetite-regulating hormone levels, body composition, and food intake over 0–6-12 months of treatment in the ICPP group (n = 20)

	Baseline	6th month	12th month	*p*
Appetite-regulating hormones				
Ghrelin (ng/mL)	8.10 (4.12–13.97)	7.37 (2.81–11.90)	7.54 (4.10–16.56)	0.216
Leptin (ng/mL)	2.79 (0.40–13.40)	4.85 (0.91–17.87)	5.67 (0.90–17.72)	0.022*
PYY (pg/mL)	46.23 (9.74–161.27)	32.05 (3.58–110.19)	32.48 (5.02–63.36)	< 0.001**
NPY (pg/mL)	2644.93 (692.18–14,349.27)	1991.42 (330.48–7999.09)	1322.13 (585.12–4325.23)	< 0.001**
Body composition analysis				
BMI SDS	1.00 ± 0.86	1.06 ± 0.96	1.15 ± 0.93	0.157
Body fat mass (kg)	7.2 (4.4–22.7)	9.6 (4.7–18.5)	10.4 (4.6–25.8)	< 0.001*
Body fat percentage (%)	23.9 (17.5–41.1)	27.4 (21.2–38.0)	28.4 (19.8–42.6)	< 0.001*
Fat-free mas (kg)	22.4 (14.1–35.9)	24.0 (14.3–36.8)	25.4 (15.3–39.6)	< 0.001*
Muscle mass (kg)	21.2 (13.3–34.1)	22.7 (13.5–34.9)	24.1 (14.5–37.6)	< 0.001*
Truncal fat mass (kg)	3.0 (1.6–10.1)	4.5 (2.2–8.2)	4.6 (1.9–11.8)	< 0.001*
Truncal fat percentage (%)	18.6 (10.6–35.6)	21.2 (14.3–32.4)	23.0 (13.6–36.2)	< 0.001*
24 h food intake record				
Total caloric intake (kcal)	1002 (818–1348)	1181 (861–1601)	1146 (886–1492)	0.077
Carbohydrate intake (gr)	116.5 (83.9–170.5)	146.2 (87.3–244.9)	162.7 (78.2–205.2)	0.448
Fat intake (gr)	37.1 (32.4–83.6)	45.8 (18.2–84)	46.2 (32–60.1)	0.232
Protein intake (gr)	34.5 (27.2–59.1)	41.6 (23.4–96.8)	34.4 (23.4–100.5)	0.053
Macronutrient ratios				
Carbohydrate (%)	50.5 (27–63)	53 (28–72)	52 (31–64)	0.187
Fat (%)	34 (26–55)	34.5 (14–54)	36 (25–52)	0.523
Protein (%)	14 (11–18)	14 (9–21)	12 (11–28)	0.060

*BMI* *SDS* body mass index standard deviation score, *ICPP* idiopathic central precocious puberty, *NPY* neuropeptide Y, *PYY* peptide YY

^*^Statistically different between baseline and the 6th month

^**^Statistically significant between baseline and the 6th month, as well as between baseline and the 12th month

To examine whether the trends in these parameters over time depend on the form of leuprolide acetate, a two-way repeated measures ANOVA was performed (logarithmic transformations were made for ghrelin, leptin, PYY, and NPY levels to obtain a parametric distribution for the analysis). The form of leuprolide acetate had no interaction effect on changes in all four appetite-regulating hormone levels at 0, 6, and 12 months of treatment (F = 1.177 p = 0.320 for ghrelin, F = 0.219 p = 0.729 for leptin, F = 0.268 p = 0.767 for PYY, and F = 1.528 p = 0.231 for NPY). Similarly, there was no interaction effect of the form of leuprolide acetate on changes in both BMI SDS and body fat percentage at 0, 6, and 12 months of treatment (F = 0.238, p = 0.789 and F = 0.038, p = 0.963, respectively). This indicates that the effects of leuprolide acetate treatment on appetite-regulating hormone levels, BMI SDS, and body composition were similar across the two forms.

### Correlations between the study parameters

**Fig. 2:**
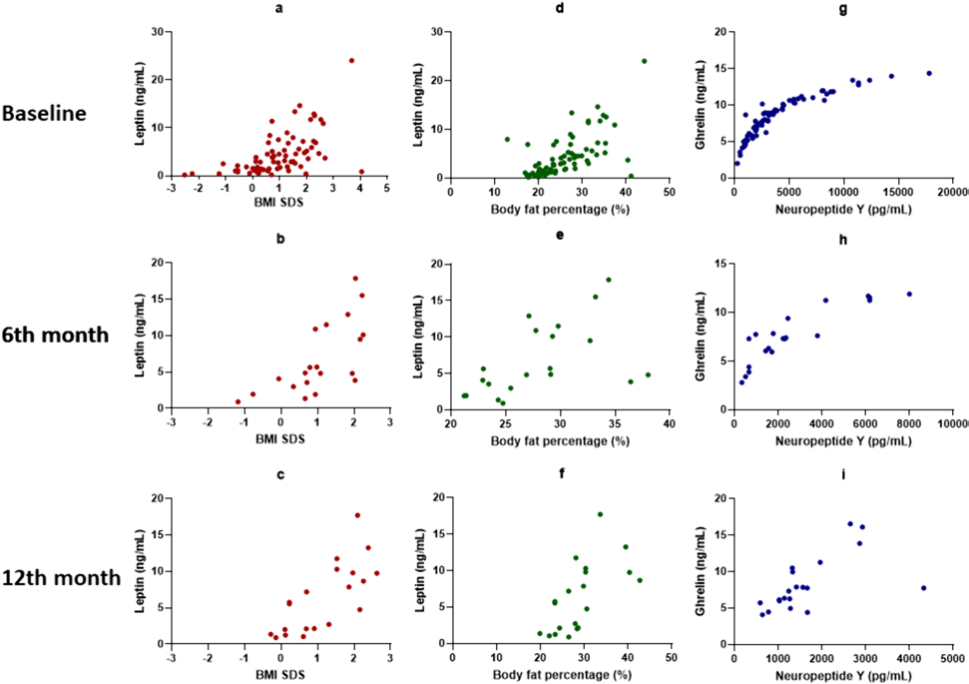
Scatter dot graphs showing the correlations between leptin and BMI SDS, leptin and body fat percentage, ghrelin and NPY at baseline for the overall group, also at the 6th and the 12th months of the treatment in the ICPP group. **a** p < 0.001, r = 0.635 **b** p < 0.001, r = 0.713 **c** p =  < .001, r = 0.778 **d** p < 0.001, r = 0.675 **e** p = 0.005, r = 0.597 **f** p = 0.001, r = 0.676 **g** p < 0.001, r = 0.967** h** p < 0.001, r = 0.882 **i** p < 0.001, r = 0.716

In the overall group, leptin levels correlated well with BMI SDS and fat percentage, and also there was a strong positive correlation between serum ghrelin and NPY levels. These correlations were also present at the 6th and 12th months of the treatment in the ICPP group (Fig. 2).

## Discussion

The first main finding of the study is that ghrelin, leptin, PYY, and NPY levels were similar between prepubertal girls and girls with ICPP. This finding proposes that there is no direct role of these hormones on the onset of puberty. The relationship between several appetite-regulating hormones and pubertal onset and development has long been investigated, mainly focusing on leptin [20, 21]. In a longitudinal study, serum ghrelin and PYY levels were measured in 26 girls from 10 to 13 years of age, and neither of these hormones showed a relationship with the pubertal stages of the girls [20]. In a larger cross-sectional study, where leptin levels were measured in 401 girls, a positive correlation between leptin levels and pubertal stages were shown. However, body fat percentage also increased with pubertal stages, along with leptin levels. Therefore, as leptin is highly related to adipose tissue, the researchers attributed the increase in leptin levels to the concomitant increase to body fat percentage rather than pubertal stages [21]. As for NPY, only one study investigated the difference in serum NPY levels between girls with precocious puberty and age-matched prepubertal girls, showing no difference at all [22]. However, it should be noted that almost all investigations on the relationship between NPY and puberty have focused on the hypothalamic levels of NPY in animal studies and not the circulating levels.

The second main finding of this study is the increase in body fat percentage despite no change in BMI SDS in the patients on GnRH treatment. As for the impact of GnRH analogue therapy on BMI and body composition, the studies show highly contradicting results. While some studies report a significant weight gain during GnRH analogue treatment, some studies report no change. In one study, it was reported that the weight gain is prominent in normal-weight girls under GnRH analogue treatment, while there is no significant weight gain in girls who already have obesity in the beginning of the therapy. In the present study, no significant alteration in BMI SDS was found; however, it should be noted that these patients were followed up in a dietitian outpatient clinic with 6-month intervals due to the prospective nature of the study. However, as a striking finding, despite no significant change in BMI SDS, fat percentage was found to increase in girls with ICPP under leuprolide acetate treatment. Although this finding has already been reported in a few studies, the authors did not propose an explanation for this. Estrogen regulates fat distribution and body adiposity through its receptors (ERα and ERβ), and the changes in the perimenopausal state, where estrogen levels decrease, akin to the girls on GnRH analogue treatment, have been well-defined: an accumulation of fat, prominently in the trunk [23, 24]. Estrogen is known to suppress the transcription of lipoprotein lipase gene [25]. Therefore, the increase in body fat under GnRH treatment might be due to decrease in estrogen levels which will result in disappearance of the suppression and activation of lipoprotein lipase gene.

As for the change in the appetite-regulating hormones during GnRH analogue therapy, very few studies have been conducted. In a recent study from Thailand, 18 girls with central precocious puberty were followed up under GnRH analogue treatment for 20 weeks, resulting in no change in serum ghrelin and PYY levels, but a significant increase in leptin levels along with fat percentage [14]. On the other hand, another study showed no change in leptin levels at the 3rd and 6th months of GnRH analogue therapy in 37 girls with central precocious puberty. However, at each milestone, leptin levels correlated positively with BMI, and the authors concluded that leptin levels were entirely associated with BMI rather than pubertal status [15]. In our study, similarly, the increase in leptin under LA treatment paralleled the significant increase in body fat percentage. This finding supports that the change in leptin primarily reflects the alteration in body fat percentage rather than pubertal status.

In this study, PYY levels showed a significant decrease under LA treatment. Based on the close relationship reported in the existing literature between body fat and PYY, similar as leptin, we believe that this decrease was caused by the changes in the body composition rather than the pubertal suppression. A negative correlation between PYY and BMI has been reported both in adults [26] and children [27]. Also, PYY levels increased after weight loss in a study conducted in children [27]. On the other hand, in a mouse experiment, PYY levels were reported to decrease in diet-induced obese mice, which was explained as a compensatory regulation to the increase in PYY receptors in the medulla [28]. Although BMI did not change in our study, the increase in body fat percentage may have caused the decrease in PYY levels.

As for NPY, no study has investigated the change in circulating NPY levels during LA treatment. To explain the significant decrease in circulating NPY levels observed during treatment, we can speculate on some possible mechanisms based on studies involving hypothalamic NPY levels. Firstly, estrogen has been shown to lead to an increase in the quantity of NPY neurons, elevated levels of NPY within pre-synaptic boutons, and enhanced release of NPY within the hippocampus. Additionally, estrogen promotes higher expression of NPY mRNA in both the hippocampus and caudate nucleus. Given this information, the decrease in estrogen levels during LA treatment may have led to an decrease in NPY levels [29]. However, this mechanism does not explain the similar NPY levels in pubertal girls as prepubertal ones who have significantly lower estrogen levels. Thus, the second mechanism, which appears more plausible, is that leptin's central action involves the reduction of NPY synthesis and a consequent decrease in NPY levels within the arcuate nucleus [30]. Therefore, the elevation of leptin levels in parallel with the increase in fat percentage may have caused the observed decrease in NPY levels.

This study is not without limitations. While our study yielded numerous significant findings, it is important to acknowledge that the relatively small sample size may have influenced the results. Additionally, our prepubertal control group lacked longitudinal follow-up. Therefore, the analysis of changes in the study parameters could only be conducted within the patient group, which introduced speculative aspects to the study. On the other hand, the comprehensive approach to the topic, including the evaluation of food intake records and body composition analysis, was the strength of the study.

## Conclusion

When the findings of the study were evaluated as a whole, it was observed that appetite-regulating hormones do not play a direct role in the pathogenesis of precocious puberty. Nevertheless, the puberty blockade was found to induce changes in the circulating levels of these hormones, leading to alterations in body composition. This clinical study can be viewed as a preliminary investigation that lays the groundwork for future molecular studies to elucidate the role of appetite-regulating hormones in precocious puberty.

## Supplementary Information

Below is the link to the electronic supplementary material.

Supplementary file1 (DOCX 16 KB)

## Data Availability

The data that support the findings of this study are available from the corresponding author upon reasonable request.
