# Supplementary material for: The role of body composition and appetite-regulating hormones in idiopathic central precocious puberty and their changes during GnRH analog therapy
Source: J Endocrinol Invest. 2024 Jun 19;48(1):145–52. doi: 10.1007/s40618-024-02413-3 (PMC11729209; doi:10.1007/s40618-024-02413-3)
Supplement: Supplementary file 1 — Supplementary file1 (DOCX 16 KB) [file 40618_2024_2413_MOESM1_ESM.docx]

02.06.2024

Dear editor,

I would like to thank all the reviewers for their meticulous review and positive comments on my work. Below are my point-by-point responses to the comments.

COMMENTS TO THE AUTHOR:

Reviewer #2: The Authors of the study aimed to compare circulating levels of ghrelin, leptin, PYY) and NPY between girls with ICPP and prepubertal girls and  to evaluate the trend of  these hormone levels and body composition during leuprolide acetate treatment in girls with ICPP.
This prospective study appears well conducted and of interest to the pediatric endocrinologist because it addresses a controversial topic for which few clinical studies are available.  However, some revisions are needed.

I have the following comments:

1)      Introduction, page 3, line 45: in my opinion , a specific reference regarding this pathology would be appropriate (i.e. von Schnurbeint et al, Horm Res Paediatr 2012)

Page 4, line 8: similarly, even if the topic is addressed in the cited review, a specific reference would also be appropriate (i.e. Miller 2011 or Misra 2006)

Page 4, line 25: the few studies in the literature are commented on in the Discussion, but it would be appropriate to at least mention them in this section

Thank you for your comments. I have added the suggested articles as citations in the relevant sections.

2)      Materials and Methods, page 4, line 42: The term "cross-sectional" does not seem correct to me, considering that patients with ICPP are included in a follow-up and examined 6 and 12 months after the start of therapy.

Thank you for your comment. Upon reviewing your feedback and re-evaluating our study design, we acknowledge that the term "cross-sectional" may not accurately describe our study as it involves following up with patients with ICPP at 6 and 12 months after the start of therapy. Initially, we considered the study cross-sectional because the inclusion of girls with premature thelarche was made within a specific date range. However, given that we analyzed follow-up data, the study aligns more closely with a prospective design. Therefore, we agree with your suggestion and have removed the term "cross-sectional" from the manuscript to more accurately reflect the study's methodology.

Page 4, line 53: in my opinion, the correct term at this point is CPP. In fact, the ICPP definition can only be validated after the MR has been performed.

Thanks for the comment. To clarify, our manuscript states in the Materials and Methods section that "Girls diagnosed with precocious puberty underwent contrast-enhanced brain and pituitary MRI. Those found to have an organic pathology were excluded from the study, leaving only idiopathic cases." Therefore, the use of "ICPP" in our manuscript is based on MRI confirmations that excluded organic causes.

Page 5, lines 4-6: the Authors should provide more information on the selection criteria for prepubertal controls with respect to the generic phrase.....(short normals? subjects referred for a suspected endocrine problem and found to be healthy ?...)

In response to your request for additional information on the selection criteria for the prepubertal controls, I would like to clarify that the controls were not recruited from the pediatric endocrinology outpatient clinic, but from the general pediatric outpatient clinic. These controls consisted of children who presented for routine check-ups or had concerns related to height, weight, or pubertal development. Upon evaluation, they were found to be free from any endocrinological disorders and did not have any chronic diseases or medication use.

3)      Results, page 7, lines 44-47: Is the trend of appetite regulating hormones different depending on the type of monthly or quarterly treatment?

Thank you for your insightful question regarding whether the trend of appetite-regulating hormones differs depending on the type of leuprolide acetate treatment—monthly versus quarterly. In response to your query, we have conducted additional analyses to explore this aspect further. The findings from these analyses have now been added to the Results section of our manuscript.

*“…To examine whether the trends in these parameters over time depend on the form of leuprolide acetate, a two-way repeated measures ANOVA was performed (logarithmic transformations were made for ghrelin, leptin, PYY, and NPY levels to obtain a parametric distribution for the analysis). The form of leuprolide acetate had no interaction effect on changes in all four appetite-regulating hormone levels at 0, 6, and 12 months of treatment (F=1.177 p=0.320 for ghrelin, F=0.219 p=0.729 for leptin, F=0.268 p=0.767 for PYY, and F=1.528 p=0.231 for NPY)...”*

Table 1. It should be specified that some parameters have been expressed as mean and SD and others as median and rangeWhat do PAH-av and PAH-ac mean? The meaning is not shown in the abbreviations list.

Thanks for the comment. Data with a parametric distribution were presented as mean±SDS, while data with a nonparametric distribution were presented as median (minimum-maximum). This statement has been added to the methods section. As for the abbreviations PAH-av and PAH-ac, we have checked our manuscript but these abbreviations do not appear in the text or tables.

Reviewer #3: Tarcin and coworkers are here illustrating the results of their study concerning the role of body composition and appetite-regulating hormones in idiopathic central precocious puberty together with their changes during GnRH analog therapy. The study is well designed and very well illustrated. Results are interesting and deserve their publication after amending minor typos errors.

-Evaluation and subjects, Page 5 Line 29: "… method. A GnRH stimulation…"
-Results, Page 7 Line 15: "..ICCP, half…"

Thanks for your positive comments. The errors have been corrected.

I hope that with these revisions, addressing the insightful feedback provided, my manuscript is now ready for acceptance. Thank you for the opportunity to enhance our manuscript through your constructive comments.
